# 89. On-going impact of the SARS-CoV-2 pandemic on the evolution of carbapenemase-producing *Enterobacterales* in Ontario, Canada

**DOI:** 10.1093/ofid/ofac492.014

**Published:** 2022-12-15

**Authors:** Mohammad Mozafarihashjin, Alainna J Jamal, Christopher Kandel, Philipp Kohler, Laura Mataseje, Michael Mulvey, Vanessa Allen, Kevin R Barker, Mahin Baqi, Sergio Borgia, Brenda Coleman, Amna Faheem, Lubna Farooqi, Jennie Johnstone, Kevin Katz, Roberto Melano, Matthew Muller, Samira Mubareka, Samir Patel, Susan Poutanen, David Richardson, Angel Li, Zoe Zhong, Allison McGeer

**Affiliations:** Sinai Health System, University of Toronto, Toronto, ON, Canada; Sinai Health System, University of Toronto, Toronto, ON, Canada; Toronto East Health Network, University of Toronto, Toronto, Ontario, Canada; Hopital St. Gallen, St. Gallen, Zurich, Switzerland; National Microbiology Laboratory, Winnipeg, Manitoba, Canada; National Microbiology Laboratory, Winnipeg, Manitoba, Canada; Sinai Health System, University of Toronto, Toronto, ON, Canada; Trillium Health Partners, Toronto, Ontario, Canada; William Osler Health System, Toront, Ontario, Canada; William Osler Health System, Toront, Ontario, Canada; Sinai Health System, University of Toronto, Toronto, ON, Canada; North York General Hospital, Toronto, Ontario, Canada; Sinai Health System, University of Toronto, Toronto, ON, Canada; University of Toronto, Toronto, Ontario, Canada; North York General Hospital, Toronto, Ontario, Canada; Public Health Ontario, Toronto, Ontario, Canada; Unity Health, University of Toronto, Toronto, Ontario, Canada; Sunnybrook Health Sciences Centre, University of Toronto, Toronto, Ontario, Canada; Public Health Ontario, Toronto, Ontario, Canada; Sinai Health System, University of Toronto, Toronto, ON, Canada; William Osler Health System, Toront, Ontario, Canada; Sinai Health System, University of Toronto, Toronto, ON, Canada; Sinai Health System, University of Toronto, Toronto, ON, Canada; Mt. Sinai Hospital, Toronto, Ontario, Canada

## Abstract

**Background:**

The spread of carbapenemase-producing *Enterobacterales* (CPE) is global threat. Numerous outbreaks of CPE have been reported during the COVID-19 pandemic. We describe the impact of of the SARS-CoV-2 pandemic on the emergence of CPE in south-central Ontario, Canada.

Incidence of clinical isolates of CPE and isolates with different CPE genes in Toronto/Peel region, 2017–2021.

The upper panel shows the incidence of patients with clinical isolates of CPE by year and quarter from q4 2007 to q1 2022. The lower panel shows the incidence of patients with clinical isolates with different carbapenemase genes by fiscal year during the same period.

**Methods:**

TIBDN has performed population-based surveillance for CPE in Toronto/Peel region (pop 4.5M) from first identified isolate in 2007. All laboratories test/refer all carbapenem non-susceptible Enterobacterial isolates for identification of CPE. Hospital charts are reviewed and patients/physicians interviewed. Population data are obtained from Statistics Canada.

**Results:**

From 10/2007 to 3/31/2022, 1367 persons colonized or infected with CPE were identified. Their median age was 68.7yrs (IQR 54–78yrs); 761 (56%) were male. 772 (56%) were colonized when first identified; 115 (8.4%) were bacteremic at identification or subsequently developed bacteremia. The most common organisms were *E. coli* (651, 48%), K*. pneumoniae* (436, 32%), *Enterobacter spp*. (146, 11%), *Citrobacter spp* (62, 5%); the most common genes were NDM±OXA-48 (722, 53%), OXA-48-like (341, 25%), KPC (225, 16%), VIM (44, 3%). The incidence of CPE infections increased steadily until 3/2020 then declined by 61% and remained stable until 3/2022 (Figure, upper panel). The decline was greater for *E. coli* (56% decrease), *K. pneumoniae* (62%) than for *Enterobacter spp.* (30%) and other species (19%). It occurred in all genes in 2020; however, KPC containing organisms increased again in 2021 (Figure, lower panel).

**Conclusion:**

The advent of the COVID-19 pandemic was associated with an immediate, substantial decline in the incidence of patients with CPE in our population area. This decline occurred in both isolates with genes usually occurring in cases imported from other countries, and in those usually occurring in cases associated with transmission within Canadian hospitals. Decreased travel and enhanced infection prevention and control in hospitals may both have contributed to reductions in CPE during the pandemic.

**Disclosures:**

**All Authors**: No reported disclosures.

